# Enhancing Wear Resistance in Functionally Graded Metallic Components: Insights from Nanoindentation and Mechanical Analysis

**DOI:** 10.3390/ma17071567

**Published:** 2024-03-29

**Authors:** Osamu Furukimi, Hitoshi Kabasawa, Masayuki Yamamoto, Roonie Protasius, Masaki Tanaka

**Affiliations:** 1 Department of Materials, Kyushu University, 744 Motooka Nishi-ku, Fukuoka 819-0395, Japan; mukouile4309@gmail.com (O.F.); protasius.roonie.286@s.kyushu-u.ac.jp (R.P.); 2 Nihon Techno Co., Ltd., 3968 Uruido Hasuda, Saitama 349-0133, Japan; 3 Yamamoto Scientific Tool Lab. Co., Ltd., 2-15-4 Sakae-cho Funabashi, Chiba 273-0018, Japan; m.yamamoto@ystl.jp

**Keywords:** nanoindentation, wear resistance, nitriding, carburising, elastic strain resistance, deformation energy

## Abstract

To manufacture metallic components with high wear resistance, treatments such as nitriding and carburising followed by quenching and tempering (NQT and CQT, respectively) are applied to various types of steel to increase the hardness (*H*) of the friction surface. However, the wear mechanism of the resulting functionally graded materials has not been fully understood. In this study, specimens of industrial 99.82% pure iron treated with NQT at 913 and 1033 K, and CQT at 1203 K, as well as hot-rolled sheets without heat treatment were examined by performing nanoindentation tests to measure changes in their *H*, reduced Young’s moduli (*E*_r_), elastic deformation energies (*W*_e_), and plastic deformation energies (*W*_p_) along the depth direction. The relationship between *W*_p_/*W*_e_ and the elastic strain resistance (*H*/*E*_r_) can be expressed for all specimens via the equation *W*_p_*/W*_e_ = −1.0 + 0.16 (*H*/*E*_r_)^−1^. Furthermore, the obtained *H*/*E*_r_ av measured at 5 µm intervals based on the specimen profile and wear volume has a good correlation depending to the sliding distance, as confirmed by the results of the ring-on-plate sliding tests conducted for the carbon-treated, nitrogen-treated, and hot-rolled specimens. This study provides a new approach, using *H*/*E*_r_ parameters to identify the dominant factors affecting wear resistance at the initial stage of wear that may contribute to the development of wear-resistant materials.

## 1. Introduction

High wear resistance is essential for enhancing the durability of materials used in mechanical structural parts. Increasing the hardness (*H*) of the friction surface considerably improves wear resistance [1]. Various coating materials and their processes have been developed to increase surface hardness [2,3,4,5,6,7,8,9,10]. In particular, the carburising–quenching–tempering (CQT) process [11,12,13], which involves heating above 1123 K in an acetylene gas atmosphere to facilitate carbon atom diffusion into steel, has been widely industrialised. Additionally, the industrial application of the nitriding–quenching–tempering (NQT) process, which involves heating to 893–1103 K in an ammonia gas atmosphere to promote nitrogen atom diffusion into the steel followed by quenching–tempering, is continuously expanding [14,15]. The thermal strain in the NQT-treated parts is lower than that in the CQT-treated parts owing to the lower heating temperature [16].

Both the coating and CQT/NQT processes aim to increase the surface hardness. However, the effect of the increased hardness on the wear resistance of the CQT-treated and NQT-treated steels with functionally graded material (FGM) properties [17,18,19,20] has not been fully elucidated. Plastic deformation properties and wear resistance have been previously analysed using the *H*/*E* ratio, where *E* is Young’s modulus. Greenwood and Williamson [21] examined the plastic deformation behaviour of specimen surfaces using a Hertzian (spherical) indenter by considering surface irregularities. They found that the plastic deformation area caused by indentation was inversely proportional to the elastic strain resistance (*H*/*E*_r_), where *E*_r_ was the reduced Young’s modulus, suggesting a higher plastic deformation degree of the specimen with a smaller *H*/*E*_r_ ratio. Leyland and Matthews [22] demonstrated the existence of a correlation between *H*/*E*_r_ and the wear resistance of nanocomposite coatings. Furthermore, Halling [23] provided numerical evidence for ceramic-coated materials, suggesting that *H*^2^/*E* is strongly correlated with stress, which determines the elastic limit under specific friction conditions.

Coated materials typically contain a uniformly hardened layer with a thickness of several micrometres on the surface, while carburised steels exhibit a hardness range of 10–100 µm [24,25,26]. Therefore, it is crucial to assess *H* and *E*_r_ at the micrometre scale and investigate the effects of changes in *H* and *H*/*E*_r_ along the depth direction on the wear resistance of CQT-treated and NQT-treated steels. Recent advances in nanoindentation testing have enabled the measurement of the total deformation energy (*W*_t_), elastic deformation energy (*W*_e_), and plastic deformation energy (*W*_p_) at the micrometre scale. Okoro et al. [27] performed nanoindentation testing to explore the effect of multiwalled carbon nanotubes (MWCNTs) on the mechanical properties of Ti_6_Al_4_V sintered materials and established correlations between *W*_e_/*W*_t_, *W*_p_/*W*_t_, *H*/*E*_r_, *H*^3^/*E*_r_^2^, and the added amount of MWCNTs.

Previously, Yamamoto et al. determined the relationship between *H*/*E*_r_ and *W*_p_/*W*_e_ for austenitic stainless steels, high-purity copper, single-crystal tungsten, and the quasi-elastically heat-treated 45mass%Ti–55mass%Ni alloy [28]. As a result, the following formula was derived:

*W*_p_/*W*_e_ = *a* + *b* (*H*/*E*_r_)^−1^ (1)

where *a* and *b* are the constants equal to at −1 and 0.16, respectively. This equation is in good agreement with the findings of Yang et al. [29] and Yamamoto et al. [28], who established a linear relationship between *H*/*E*_r_ and *W*_e_/*W*_t_ (Equation (2)). Moreover, the relationship between *H*/*E*_r_ and *W*_p_/*W*_t_ determined by Cheng and Cheng [30] and Yamamoto et al. [28] is expressed by Equation (3).

*H*/*E*_r_ = α (*W*_e_/*W*_t_) (2)

*H*/*E*_r_ = β + γ (*W*_p_/*W*_t_) (3)

Note that Equation (1) can be obtained by eliminating *W*_t_ from Equations (2) and (3). The parameter *W*_p_/*W*_e_ indicates the effect of the deformation energy on the elastic limit and subsequent plastic deformation energy. In addition, Yamamoto et al. [31] concluded that Equation (1) did not depend on the maximum load during nanoindentation testing. These findings suggest that both *H*/*E*_r_ and *W*_p_/*W*_e_ are valuable parameters for evaluating the wear resistance and the physical interpretation of hardness.

The effect of nitriding conditions during the NQT treatment of 99.82% pure iron on its microstructure was examined previously [24]. Nitriding at 913 K for 5.4 ks generated an iron nitride phase on the steel surface, whereas nitriding at 1033 K produced high-nitrogen martensite. Both surface layers exhibited higher wear resistances as compared with those of the untreated hot-rolled specimens under the no-lubrication condition in a ring-on-plate sliding test. In another study [25], a similar wear resistance was obtained for pure iron treated via NQT and CQT. However, a specific way to estimate the wear resistance has not been established yet in FGMs. Therefore, we anticipate that the *H*/*E*_r_ and *W*_p_/*W*_e_ parameters obtained via nanoindentation testing can provide further insights into this phenomenon.

In this study, the distributions of *H*/*E*_r_ and *W*_p_/*W*_e_ on the surfaces of the specimens of 99.82% pure iron treated via NQT and CQT as well as the untreated (as hot-rolled) specimens were investigated by performing nanoindentation testing. The obtained relationships were compared with those of other materials, including steels, high-purity copper, single-crystal tungsten, and the quasi-elastically heat-treated 45mass%Ti–55mass%Ni alloy [28]. Finally, the wear resistance was discussed, conducting a ring-on-plate sliding test under the non-lubricated condition. The relationships between the wear resistance and *H*/*E*_r_ and *W*_p_/*W*_e_ obtained with the nanoindentation tests were also discussed.

This work proposes a new approach using the elastic and plastic energies determined via nanoindentation testing to examine the wear resistance of metallic materials. Its results can help us to achieve a better understanding of FGM mechanical properties at the microscale.

## 2. Experimental Procedures

### 2.1. Specimens and Microstructural Observations

The chemical composition and heat treatment conditions of the industrial pure iron are presented in Table 1 and Table 2, respectively. Nitriding was performed at 913 K (NQT913) and 1033 K (NQT1033), whereas the carburising procedure involved heating to 1203 K followed by a carbon diffusion treatment at 1113 K (CQT1203). The conditions of the heat treatment are those used in industry. Carbon diffusion treatment was conducted in order to prevent excess carburising where the surface is too hard for the application. Nitrogen molecules are not absorbed into the specimens, so the nitriding is negligible under this condition. Additionally, a hot-rolled specimen (No HT) was studied for comparison.

For microstructural and EBSD analyses, the fabricated specimens were cut cross-sectionally in the rolling direction and polished. The polishing process involved the use of emery papers with grit sizes ranging from #340 to #2000 followed by polishing with an alumina suspension (grain size: 0.3 μm) and colloidal silica. The polished specimens were etched using a 3% Nital solution. Microstructural observations were conducted using a field-emission scanning electron microscope (FE–SEM, Ultra-55, Carl Zeiss, Oberkochen, Germany) at an accelerating voltage of 5 kV. For a more detailed analysis, electron backscattered diffraction was employed in addition to FE–SEM at an accelerating voltage of 20 kV.

### 2.2. Nanoindentation Testing

In this study, the changes in *H*, *H*/*E*_r_, *W*_p_, and *W*_e_ were measured along the cross-sections of the fabricated specimens via nanoindentation testing. The specimens were first embedded in epoxy and then subjected to wet polishing with emery papers up to 2000 grit. This procedure was followed by mirror polishing using diamond abrasive and colloidal silica to prepare the surface for testing.

The *H*, *E*_r_, *W*_e_, and *W*_p_ parameters were determined from the load–displacement curves recorded using a nanoindentation tester (ENT-2100, Elionix, Musashino City, Japan). Testing was conducted at a maximum load (*F_max_*) of 9.8 mN and loading rate of 0.98 mN/s using a Berkovich-type indenter. *H* and *E*_r_ were calculated via Equations (4) and (5) [32], respectively.

(4) $$H=F_{max}/A_{p}(h_{c})$$

(5) $$E_{r}=\sqrt{\pi }/(2C\sqrt{A_{p}\left(h_{c}\right)})$$

where *A*_p_(*h*_c_) represents the projected contact area, and C is the compliance of the contact. *W*_e_ and *W*_p_ were determined according to the ISO14577-1 standard [32]. Their values were automatically calculated using the software supplied with the nanoindentation tester. For each specimen, 30 measurement points were examined at the same depth.

### 2.3. Wear Resistance Testing

A ring-on-plate-sliding tester was used to evaluate the wear resistance of the prepared specimens with dimensions of 10 mm × 10 mm × 1.2 mm (thickness). A ring with an outer diameter of 4 mm and inner diameter of 2 mm was fabricated from JIS SKD11 steel (670 HV). The ring contact surface was finished using #1000 grit emery paper.

Sliding tests were conducted at a rotational speed of 100 rpm, corresponding to a sliding speed of 31.4 mm/s under a contact pressure of 1.06 MPa without lubrication. The total sliding distance was set to 753.6 m. The test was intermittently stopped at predetermined distances of 94.2, 188.4, and 376.8 m to assess wear progression. After each stop, depth profiles of the wear scars were obtained using a 3D laser microscope (VK-9700, Keyence, Osaka City, Japan). The wear volume (*V*) was calculated from the wear profiles using a methodology described elsewhere [24].

## 3. Results

### 3.1. H, H/E_r_, and W_p_/W_e_ Distributions 

**Figure 1:**
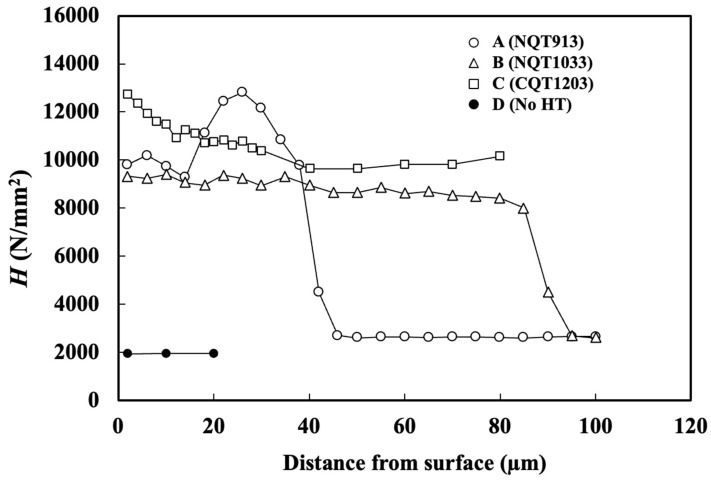
*H* profiles obtained in the directions perpendicular to the surfaces of the specimens fabricated by nitriding, quenching from 913 K, and tempering in (A); nitriding, quenching from 1033 K, and tempering in (B); CQT in (C); and without heat treatment in (D) [24,25].

The *H* profiles obtained in the direction perpendicular to the specimen surface are shown in Figure 1 [24,25]. The near-surface *H* (2 µm from the surface) of NQT913 is slightly higher than that of NQT1033. For NQT913, *H* increases up to a maximum of 12,800 N/mm^2^ at a distance of 26 µm from the surface and then decreases to a constant value of 2600 N/mm^2^ (the hardness of the base metal) at a depth of 46 µm. In contrast, the *H* of NQT1033 gradually decreases until reaching a depth of 85 µm, followed by a sharp drop to the hardness of the base metal at a depth of 95 µm.

The near-surface *H* value of CQT1203 (12,700) is greater than that of NQT913 (9800) and NQT1033 (9300). It exhibits a gradual decrease to a depth of 40 µm, ultimately reaching a constant value of 9800 N/mm^2^ [25], which is attributed to the carbon diffusion treatment (1113 K × 1.8 ks). The *H* of the No HT specimen is consistently lower than those of the heat-treated specimens, maintaining a constant value of 2000 N/mm^2^ near the surface. Note that the hardness of CQT1203 was measured up to a depth of 80 µm from the surface, as the depth of the wear track was approximately no more than 50 µm, as shown later.

**Figure 2:**
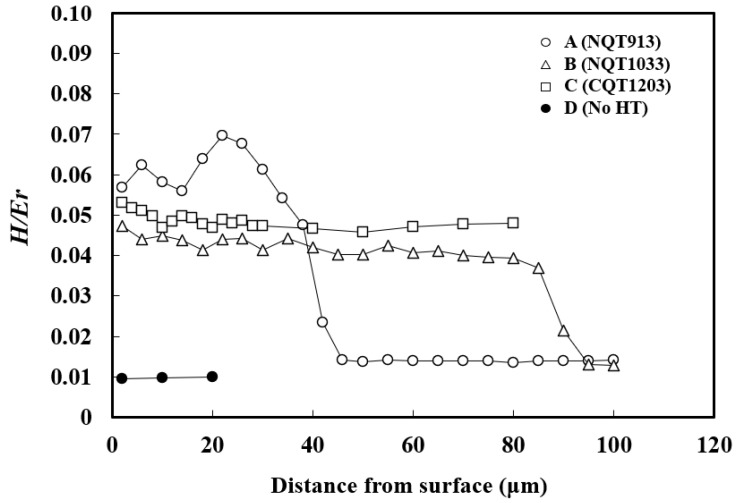
*H*/*E*_r_ profiles obtained along the directions perpendicular to the surfaces of the specimens fabricated by nitriding, quenching from 913 K, and tempering in (A); nitriding, quenching from 1033 K, and tempering in (B); CQT in (C); and without heat treatment in (D).

The *H*/*E*_r_ profiles obtained in the directions perpendicular to the specimen surfaces are shown in Figure 2. Their trends are similar to those of the *H* profiles, although with different wave amplitudes and ranks at the same distances from the surface. The near-surface *H* values decrease in the order of CQT1203 (12,700), NQT913 (9800), NQT1033 (9300), and No HT (2000). However, the near-surface *H*/*E*_r_ values decrease in the order of NQT913 (0.057), CQT1203 (0.053), NQT1033 (0.047), and No HT (0.01), indicating that the wear resistance differently correlates with *H* and *H*/*E*_r_. The relationship between the *H* or *H*/*E*_r_ value and wear volume will be shown later.

For NQT913, *H*/*E*_r_ reaches a maximum value of 0.069 at a depth of 22 µm and then rapidly decreases to a constant value of 0.014 near the base metal at 46 µm. The *H*/*E*_r_ of NQT1033 exhibits a gradual decrease to a depth of 85 µm and then rapidly decreases to a constant value at the base metal depth of 95 µm. The *H*/*E*_r_ of CQT1203 is 0.053 near the surface, assumes an intermediate value between NQT913 and NQT1033, and decreases slowly with the specimen depth up to 46 µm. The *H*/*E*_r_ ratio obtained for No HT is nearly constant of 0.01, which is lower than those of the heat-treated specimens.

**Figure 3:**
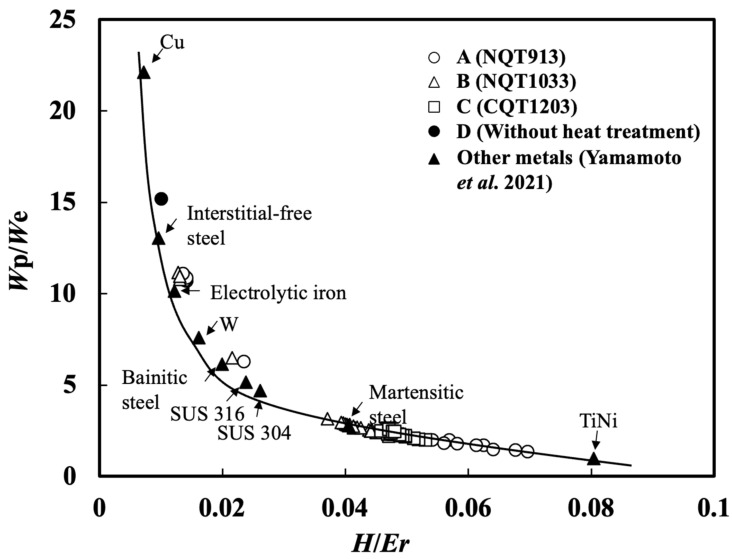
*W*_p_/*W*_e_ plotted as a function of *H*/*E*_r_. ▲ plots were from other metals [28].

In a previous study [28], a strong correlation between *W*_p_/*W*_e_ and *H*/*E*_r_ was established by performing nanoindentation tests for austenitic stainless steel, 99.99% pure copper, 99.99% pure single-crystal tungsten, and the quasi-elastic heat-treated 45mass%Ti–55mass%Ni alloy. In this work, the results obtained for the NQT913, NQT1033, CQT1203, and No HT specimens were combined with the previously obtained data (Figure 3). A strong correlation is observed between the *W*_p_/*W*_e_ and *H*/*E*_r_ values, which can be described by the following formula [28]:

*W*_p_/*W*_e_ = −1 + 0.16 (*H*/*E*_r_)^−1^ (6)

This equation demonstrates the consistency of the relationship between these two parameters for different types of materials and treatment processes.

### 3.2. Microstructural Changes along the Direction Perpendicular to the Specimen Surfaces

**Figure 4:**
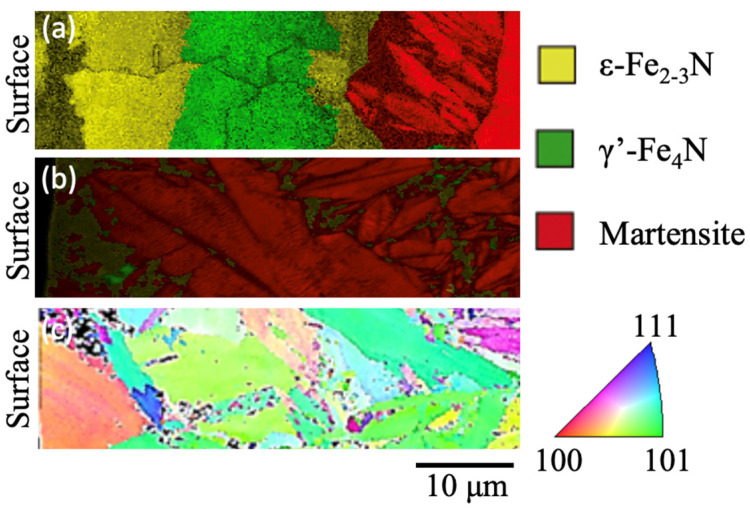
(**a**,**b**) are phase maps of the microstructure just beneath the top surface for NQT913 and NQT1033, respectively. Inverse pole figure map of CQT is shown in (**c**) [24].

**Figure 5:**
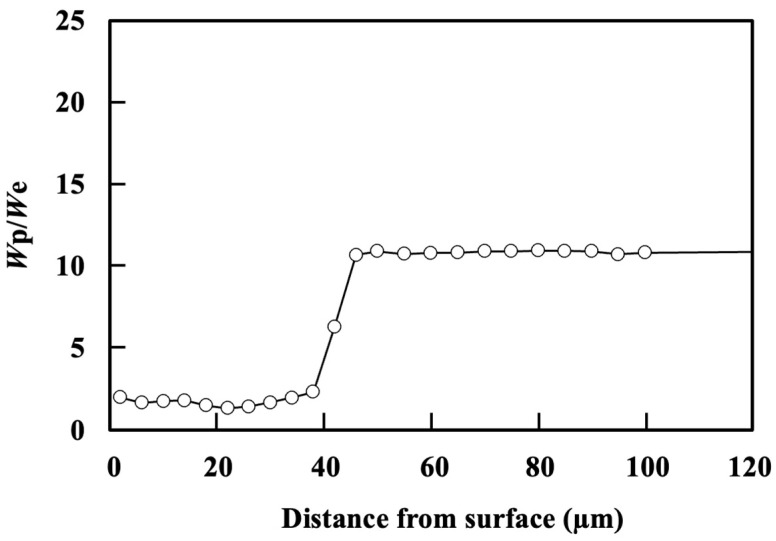
*W*_p_/*W*_e_ along the direction perpendicular to the surface of the specimen fabricated by nitriding at 913 K, quenching, and tempering [24].

**Figure 6:**
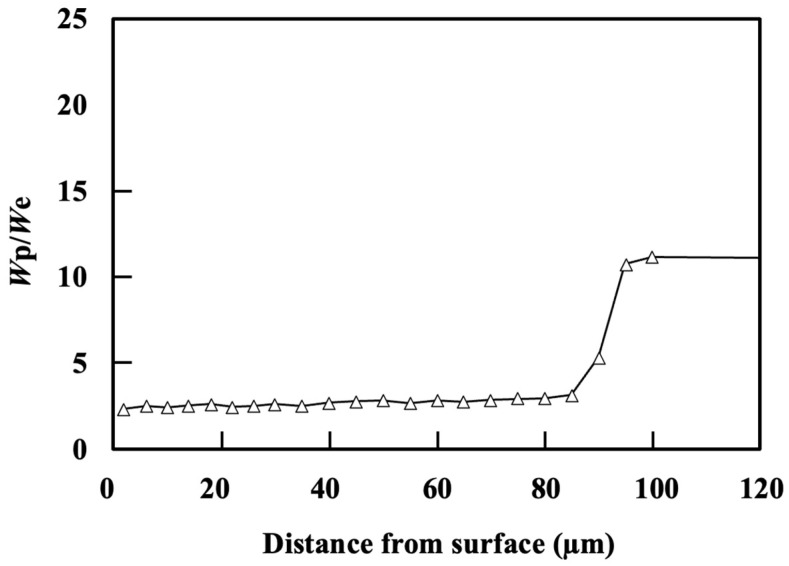
*W*_p_/*W*_e_ along the direction perpendicular to the surface of the specimen fabricated by nitriding at 1033 K, quenching, and tempering [24].

Figure 4 shows the cross-section of the microstructure beneath the specimen for the NQT913, NQT1033, and CQT1203 specimens. The details of the analyses with EPMA have been reported in a previous study [24]. The microstructure of NQT913 changes from hexagonal close-packed (hcp) (ε-Fe_2–3_N) to face-centred cubic (fcc) (γ’-Fe_4_N), followed by a body-centred cubic (bcc) (martensite) phase along the depth direction. Notably, *W*_p_/*W*_e_ exhibits its lowest value for the γ’-Fe_4_N phase and gradually increases for the martensite. In contrast, The microstructure of NQT1033 is predominantly bcc (martensite) with a minor amount of the fcc phase (retained austenite) [24]. Figure 5 and Figure 6 show that *W*_p_/*W*_e_ also demonstrates a gradual increase in the martensite phase. However, a comprehensive analysis of the effects of these microstructures on *W*_p_/*W*_e_ is a topic for future studies. Additionally, the near-surface microstructures of the CQT1203 and No HT specimens primarily consist of the martensite and ferrite phases, respectively.

### 3.3. Wear Resistance 

**Figure 7:**
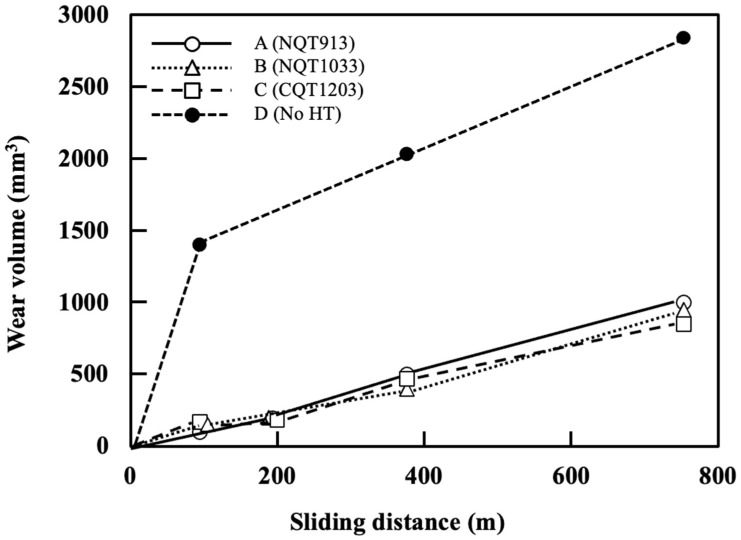
Relationships between the wear volume and sliding distance established for the specimens fabricated by nitriding, quenching from 913 K, and tempering (A); nitriding, quenching from 1033 K, and tempering (B); CQT (C); and without heat treatment (D) [24,25].

**Figure 8:**
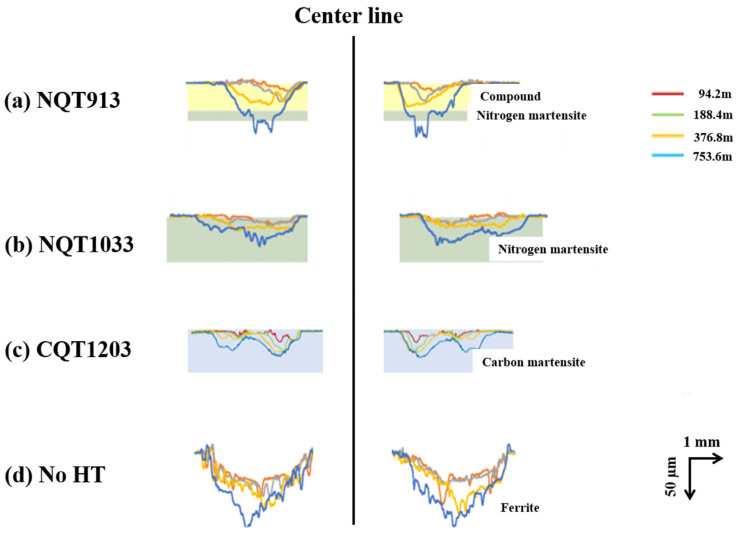
Wear profile changes with the sliding distance observed for the specimens fabricated by nitriding, quenching from 913 K, and tempering in (**a**); nitriding, quenching from 1033 K, and tempering in (**b**); CQT in (**c**); and without heat treatment in (**d**) [24,25]. Red, green, yellow, and blue lines are line profiles at the sliding distance of 92.4, 188.4, 376.8, and 753.6 m, respectively.

The correlation between the wear distance and wear volume determined by conducting ring-on-plate sliding tests is presented in Figure 7 [24,25]. Pure iron (No HT) shows relatively low hardness, so the initial amount of wear is high. However, as the test progresses, the amount of wear decreases due to the self-lubrication of the wear debris. Figure 8 shows the depth profile of the wear scar measured via laser microscopy (note that the scales of the *X*-axis and *Y*-axis in this figure are different) [24,25]. These results indicate that the wear resistance significantly increases after the NQT and CQT treatments. At a sliding distance of 376.8 m, the wear volumes decrease in the order of No HT, NQT913, CQT1203, and NQT1033. However, at the maximum tested distance of 753.6 m, the order changes to No HT, NQT913, NQT1033, and CQT1203. This result indicates that the wear resistance of the FGMs estimated only from *H* on the surface is not always accurate.

Figure 8 also shows that the width of the wear scar, which is indicative of surface plastic deformation, is smaller for NQT913 than for NQT1033, CQT1203, and No HT. Furthermore, the width-to-depth ratios of the wear scar obtained at a distance of 753.6 m for NQT913 and No HT samples are 40 and 42, respectively, whereas the values determined for NQT1033 and CQT1203 are equal to 90. These findings highlight the complex nature of the wear behaviour of these materials and will be further discussed in the following section.

## 4. Discussion

**Figure 9:**
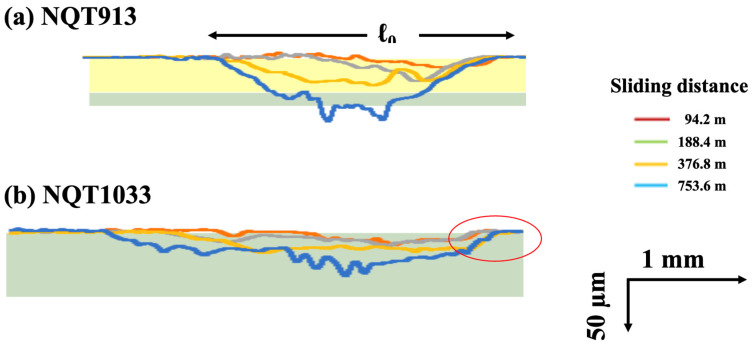
Left portions of the depth profiles of the wear scars were taken from Figure 8 for the specimens fabricated by nitriding–quenching from 913 K, and tempering (**a**); and nitriding–quenching from 1033 K, and tempering in (**b**), changing the *X*-axis and *Y*-axis scales. Red, green, yellow, and blue lines are line profiles at the sliding distance of 92.4, 188.4, 376.8, and 753.6 m, respectively.

To analyse the wear behaviour of the iron specimens more comprehensively, the left portions of the depth profiles of the wear scars obtained in Figure 8 are taken for NQT913 and NQT1033 by adjusting the scales of the *X*-axis and *Y*-axis. The obtained results presented in Figure 9 led to the following conclusions.

1. A noticeable difference in the plastic deformation at the surface (denoted as ℓ_0_) is observed between NQT913 and NQT1033.
2. The depth profile of the wear scar does not replicate the ring shape.

The first conclusion indicates that the anisotropy between the elastic and plastic deformations at the surface is a critical factor for elucidating the wear mechanism. The second observation suggests that factors other than plastic deformation (such as peeling) significantly influenced the wear process in the present study [24].

**Figure 10:**
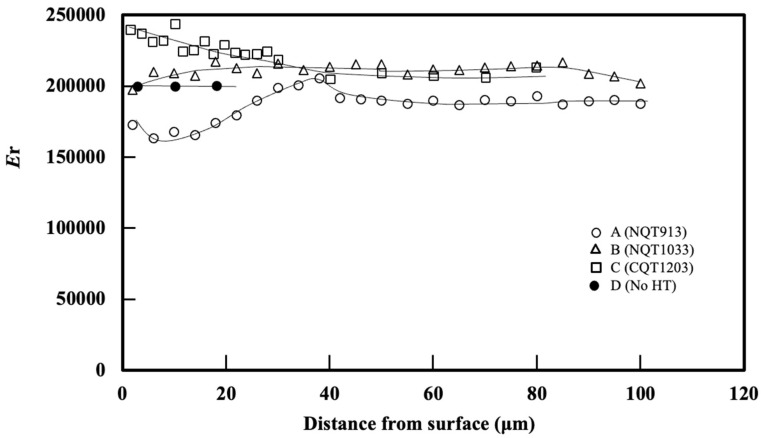
*E*_r_ profiles obtained in the directions perpendicular to the surfaces of the specimens fabricated by nitriding, quenching from 913 K, and tempering in (A); nitriding, quenching from 1033 K, and tempering in (B); CQT in (C); and without heat treatment in (D).

Next, the anisotropy between the elastic and plastic deformation will be discussed. Figure 10 shows the change in *E*_r_ observed in the directions perpendicular to the surfaces of NQT913, NQT1033, CQT1203, and NoHT. NQT1033 exhibits a continuous martensite and austenite phase to a depth of 80 µm before reaching the ferrite phase in the base metal, as indicated by the constant *E*_r_ value. In contrast, NQT913 undergoes a microstructural transition from ε-Fe_2–3_N to γ′-Fe_4_N and then to martensite with the changes in *E*_r_. Additionally, the values of *E*_r_ for the CQT and No HT samples remain nearly constant over the region spanning from the surface to the base metal. 

**Figure 11:**
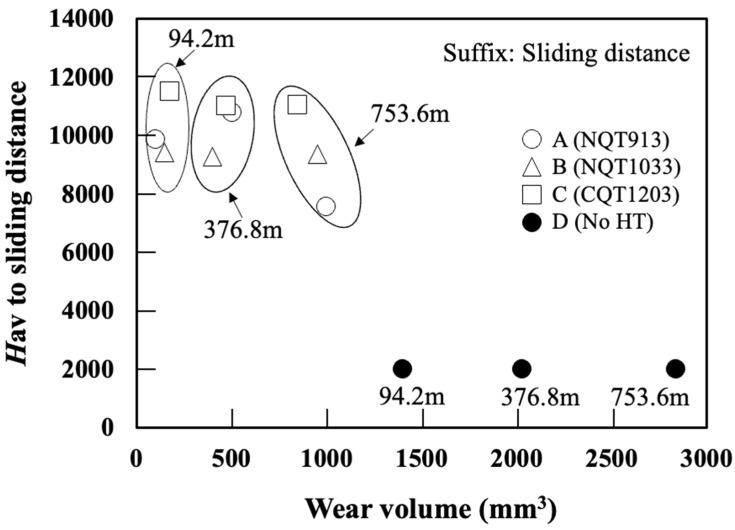
*H* av versus wear volume observed for the specimens fabricated by nitriding, quenching from 913 K, and tempering in (A); nitriding, quenching from 1033 K, and tempering in (B); CQT in (C); and without heat treatment in (D).

**Figure 12:**
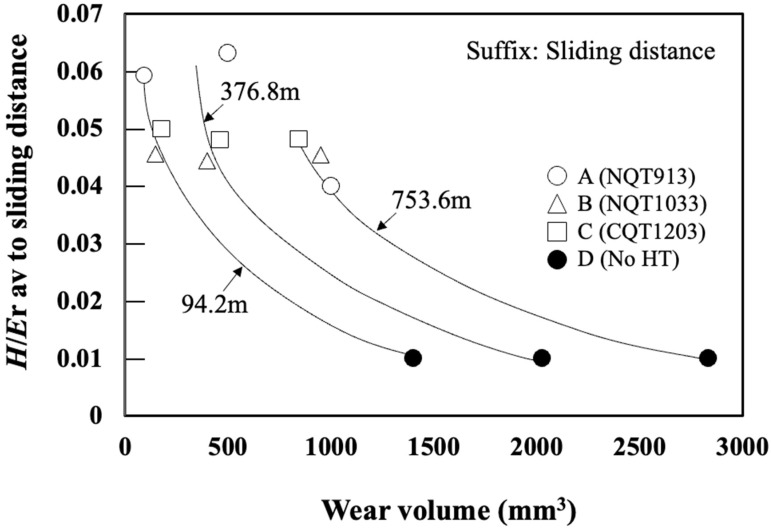
*H*/*E*_r_ av. versus wear volume observed for the specimens fabricated by nitriding, quenching from 913 K, and tempering; nitriding, quenching from 1033 K, and tempering; CQT; and without heat treatment.

Figure 11 shows the relationship between the wear volume and average of hardness (*H* av) obtained at the wear distances of 94.2, 376.8, and 753.6 m. Here, *H* av is the mean *H* value from the surface to the slid depth measured at 5 µm intervals based on the specimen profile. It suggests that the wear volume does not agree with the average hardness in any sliding distance. Figure 12 shows the relationship between the wear volume and average *H*/*E*_r_ (*H*/*E*_r_ av) obtained at the wear distances up to 94.2, 376.8, and 753.6 m. *H*/*E*_r_ av av is the mean *H*/*E*_r_ value from the surface to the slid depth measured at 5 µm intervals based on the specimen profile. Here, *H*/*E*_r_ av is calculated as the mean *H*/*E*_r_ value beneath the surface at 5 µm intervals along the cross section at the sliding distances of 94.2, 376.8, and 753.6 m measured, based on the specimen profile. For the No HT sample, which maintains a uniform microstructure along the depth direction, *H*/*E*_r_ av is the average value obtained for three measurement points.

Their relationship is in good agreement, especially at a sliding distance of 94.2 m, which is where the initial stage of wear. The trend becomes more significant as the sliding distance becomes longer. It is presumed that the wear in this regime is somewhat attributed to fractures. This was expected from the line profile of the wear track shown in Figure 8 and Figure 9. That is, the wear track of NQT1033 at the sliding distance of 753.6 m is broader and shallower than that of NQT913. This is due to the fracture of Fe_4_N within the specimen of NQT1033. Therefore, it seems there are no clear dependencies and connections between the analysed parameters, as the microstructure changes when the wear track becomes deeper. Hanse, it should be stressed that the analysed parameters are in good agreement with the initial stage of wear in functionally graded materials. 

## 5. Conclusions

This study examined the changes in *H*, *H*/*E*_r_, *W*_p_, and *W*_e_ along the cross-sections of 99.82% pure iron specimens, which were subjected to NQT at 913 (NQT913) and 1033 K (NQT1033) and CQT at 1203 K (CQT1203). A hot-rolled specimen without heat treatment (No HT) was also investigated for comparison. The analysis procedure included nanoindentation tests, and the wear resistances of the studied specimens were determined by performing ring-on-plate sliding tests without lubrication. The main conclusions from the obtained results are summarised below.

1. A strong correlation between *H*/*E*_r_ and *W*_p_/*W*_e_ was observed along the depth direction for all the specimens, which was described by the formula *W_p_*/*W_e_* = −1 + 0.16 (*H*/*E*_r_)^−1^, previously obtained for steels (including stainless steel), high-purity copper, high-purity single-crystal tungsten, and NiTi alloys.
2. The surface microstructure of NQT913 was ε-Fe_2–3_N, which transformed to γ′-Fe_4_N and then to martensite with increasing depth. In contrast, both NQT1033 and CQT1203 maintained the martensite phase at all depths, while the No HT sample contained ferrite.
3. The wear scar width of NQT913 indicating surface plastic deformation was smaller than that of the NQT1033, CQT1203, and No HT samples.
4. The wear volume at a sliding distance of 376.8 m decreased in the order of No HT, NQT913, CQT1203, and NQT1033; however, at a sliding distance of 753.6 m (the maximum distance tested), it decreased in the order of No HT, NQT913, NQT1033, and CQT1203. These results suggest that the wear resistance ranking for the carbon-treated and nitrogen-treated specimens varies with the sliding distance.
5. The average *H*/*E*_r_ av measured beneath the specimen surface displayed a good correlation with the wear volume at the initial stage of the wear.

This study describes a new approach for identifying the dominant factors affecting wear resistance that may facilitate the development of wear-resistant materials and help achieve a better understanding of FGM mechanical properties at the microscale. The futured work is a clarification of the usefulness of the new approach compared to other shapes of counterpart material used in sliding tests, such as ball-on-disk and pin-on-disk types.

## Figures and Tables

**Table 1:** Chemical compositions of the tested specimens (mass%).

C	Si	Mn	P	S	Al	N	Fe
0.003	0.001	0.16	0.011	0.004	0.001	0.0017	Bal.

**Table 2:** Heat treatment conditions used for the prepared specimens.

Specimen		Temperature (K) × Time (ks)
A	NQT913	913 K × 1.8 ks in N_2_→ 913 K × 5.4 ks in (N_2_ + NH_3_) (*K*_N_ = 0.02): Oil quenching
B	NQT1033	1033 K × 1.8 ks in N_2_ (Pressure in furnace = 30 Pa) 1033 K × 5.4 ks in (N_2_ + NH_3_) (*K*_N_ = 0.02): Oil quenching
C	CQT1203	1203 K × 3.6 ks in C_2_H_2_→ 1113 K × 1.8 ks in N_2_ (C Diffusion): Oil quenching
D	N_O_ HT	Without heat treatment

A and B were followed by tempering treatment for 5.4 ks at 533 K. C was followed by tempering treatment for 7.2 ks at 443 K. *K*_N_ is the nitriding potential, which is defined as $K_{N}=P_{NH_{3}}/{P_{H_{2}}}^{2}$, where $P_{NH_{3}}$ and $P_{H_{2}}$ are partial pressures of NH_3_ and H_2,_ respectively.

## Data Availability

Data are contained within the article.

## Nomenclature

*H*	Hardness
K_N_	Nitriding potential
NQT	Nitriding–quenching–tempering
CQT	Carburising–quenching–tempering
NoHT	Hot-rolled specimen
FGM	Functionally graded material
*E*	Young’s modulus
*E*_r_	Reduced Young’s modulus
*H*/*E*_r_	Elastic strain resistance
*W*_t_	Total deformation energy
*W*_e_	Elastic deformation energy
*W*_p_	Plastic deformation energy
MWCNTs	Multiwalled carbon nanotubes
Near-surface *H*	*H* at 2 µm from the surface
fcc	Face-centred cubic
bcc	Body-centred cubic
hcp	Hexagonal close-packed
*H* av	Mean *H* value from the surface to the sliding depth measured at 5 µm intervals based on the specimen profile
*H*/*E*_r_ av	Mean *H*/*E*_r_ value from the surface to the sliding depth measured at 5 µm intervals based on the specimen profile
*W*_p_/*W*_e_ av	Mean *W*_p_/*W*_e_ value from the surface to the sliding depth measured at 5 µm intervals based on the specimen profile
